# Supplementary material for: VDAC1 Silencing in Cancer Cells Leads to Metabolic Reprogramming That Modulates Tumor Microenvironment
Source: Cancers (Basel). 2021 Jun 7;13(11):2850. doi: 10.3390/cancers13112850 (PMC8201394; doi:10.3390/cancers13112850)
Supplement: Supplementary file 1 [file cancers-13-02850-s001.zip › cancers-1184312-supplementary.pdf]

# Supplementary Material: VDAC1 Silencing in Cancer Cells Leads to Metabolic Reprogramming That Modulates the Tumor Microenvironment

**Erez Zerbib, Tasleem Arif, Anna Shteinfer-Kuzmine, Vered Chalifa-Caspi and Varda Shoshan-Barmatz**

**Text S1: Methods**

Here, we present the complete obtained data that in the form of tables with selected representative results were presented in the main article in the form of figures. Briefly, the study was designed to address the relationship between tumor metabolism and the tumor microenvironment (TME), we used siRNA specifically with human VDAC1, as shown previously and here, that it disrupts cancer energy and metabolism homeostasis and induces metabolic reprogramming in the cancer cells derived from human cells. This allows us to follow how metabolic reprograming of cancerous affects the properties of non-cancerous cells (TME) within the tumor. To explore the interplay between metabolic reprograming of cancer cells and non-cancerous cells representing the TME within the tumor, we performed a next-generation sequencing (NGS) analysis of human A549 cell-derived tumors in a mouse model treated with non-targeting si-RNA (si-NT) or with siRNA specifically targeting human VDAC1 (si-hVDAC1−2A). As the NGS analysis allows for distinguishing between genes of human and mouse origin, we were able to demonstrate the tumor–host interactions in lung cancer.

*Determination of Mitochondrial Membrane Potential and of Cellular ATP Levels*

Mitochondrial membrane potential (Ψ) was determined using tetramethylrhodamine methylester (TMRM) dye. A549 cells were transfected with si-NT or si-hVDAC1 and 48 or 72 h post-transfection, were incubated with TMRM (700 nM, 30 min) and washed with PBS. TMRM fluorescence was measured with an Infinite M1000 plate reader. FCCP served as a control for Ψ dissipation.

Cellular ATP levels were estimated using a luciferase-based assay (CellTiter-Glo, Promega). A549 cells were transfected with si-NT or si-hVDAC1 and 36 h post-transfection were washed twice with PBS and seeded in 96-well plates at densities of $5 \times 10^4$ cells/ml. ATP levels were assayed according to the manufacturer's protocol and luminescence was recorded using an Infinite M1000 plate reader (Tecan, Männedorf, Switzerland).

**Table S1.** Antibodies used in this study.

| Antibody | Source and Cat. No. | Dilution | | |
|---|---|---|---|---|
| - | - | IHC | WB | IF |
| Mouse monoclonal anti-ATP synthase 5a | Abcam, Cambridge, UK, ab14748 | 1:300 | 1:1000 | - |
| Mouse monoclonal anti-Actin | Millipore, Billerica, MA, MAB1501 | - | 1:40000 | - |
| Rabbit polyclonal anti-Citrate Synthase | Abcam, Cambridge, UK ab96600 | 1:200 | 1:4000 | - |
| Rabbit polyclonal anti-CD31 | Abcam, Cambridge, UK ab28364 | 1:300 | | - |
| Rabbit monoclonal anti-GLUT1 | Abcam, Cambridge, UK ab40084 | 1: 200 | 1: 1500 | - |
| Mouse monoclonal anti-GAPDH | Abcam, Cambridge, UK, ab9484 | 1: 200 | 1:1000 | - |
| Rabbit monoclonal anti-HK-I | Abcam, Cambridge, UK, ab150423 | - | - | 1:200 |
| Rabbit monoclonal anti-LDH | Abcam, Cambridge, UK, ab52488 | - | - | 1:300 |

| | | | | |
|---|---|---|---|---|
| Rabbit monoclonal anti-Tenascin C | Abcam, Cambridge, UK, ab108930 | - | - | 1:100 |
| Rabbit anti-Periostin | Abcam, Cambridge, UK, ab14041 | - | - | 1:200 |
| Rabbit monoclonal anti-VDAC1 | Abcam, Cambridge, UK, ab154856 | 1:500 | 1:5000 | 1:500 |
| Goat anti-Rabbit (HRP) | KPL, Gaithersburg, USA, 474−1506 | 1:250 | 1:15,000 | - |
| Goat anti-Mouse (HRP) | Abcam, Cambridge, UK, ab97040 | 1:250 | 1:10,000 | - |
| Donkey anti-Mouse (Alexa Fluor 488) | Abcam, Cambridge, UK, ab150109 | - | - | 1:500 |
| Goat anti-Rabbit IgG H&L (Alexa Fluor 555) | Abcam, Cambridge, UK, ab150086 | - | - | 1:500 |
| Donkey anti-Mouse (Alexa Fluor 555) | Abcam, Cambridge, UK, ab150110 | - | - | 1:500 |
| Goat anti-Rabbit IgG H&L (Alexa Fluor 488) | Life technology, A−11008 | - | - | 1:500 |

Antibodies against the indicated protein, their catalogue number, source and the dilutions used in immunohistochemistry (IHC), immunofluorescence (IF) and immunoblot are presented.

**Table S2.** Real-time PCR primers used in this study.

| Gene | Primer Sequences |
|---|---|
| *COL4A1* | Forward 5′- GGCCCTTCATTAGCAGGTGT -3′<br>Reverse 5′- GTGAGGACCAACCGTTAGGG -3′ |
| *COL5A1* | Forward 5′- GATGGCGAATACTGGGTCGAT -3′<br>Reverse 5′- CCAAGAAGTGATTCTGGCTCCCT -3′ |
| *LOX* | Forward 5′- CCCAGCCACATAGATCGCAT -3′<br>Reverse 5′- CGGGAGACCGTACTGGAAGT -3′ |
| *MMP2* | Forward 5′- AACGGTCGGGAATACAGCAG -3′<br>Reverse 5′- GTAAACAAGGCTTCATGGGGG -3′ |
| *POSTN* | Forward 5′- CCCGCAGTGATGCCTATTGA -3′<br>Reverse 5′- CTCCCAAGCCTCGTTACTCG -3′ |
| *PLOD2* | Forward 5′- AGAGATATGACCTTACAAAGGGAAA -3′<br>Reverse 5′- GCAGTTGATATCAGCCGTCCA -3′ |
| *TGFB2* | Forward 5′- AAAATCGACATGCCGTCCCA -3′<br>Reverse 5′- ATGGCATCAAGGTACCCACAG -3′ |

Names of the genes examined and forward and reverse sequences of the primers used are indicated.

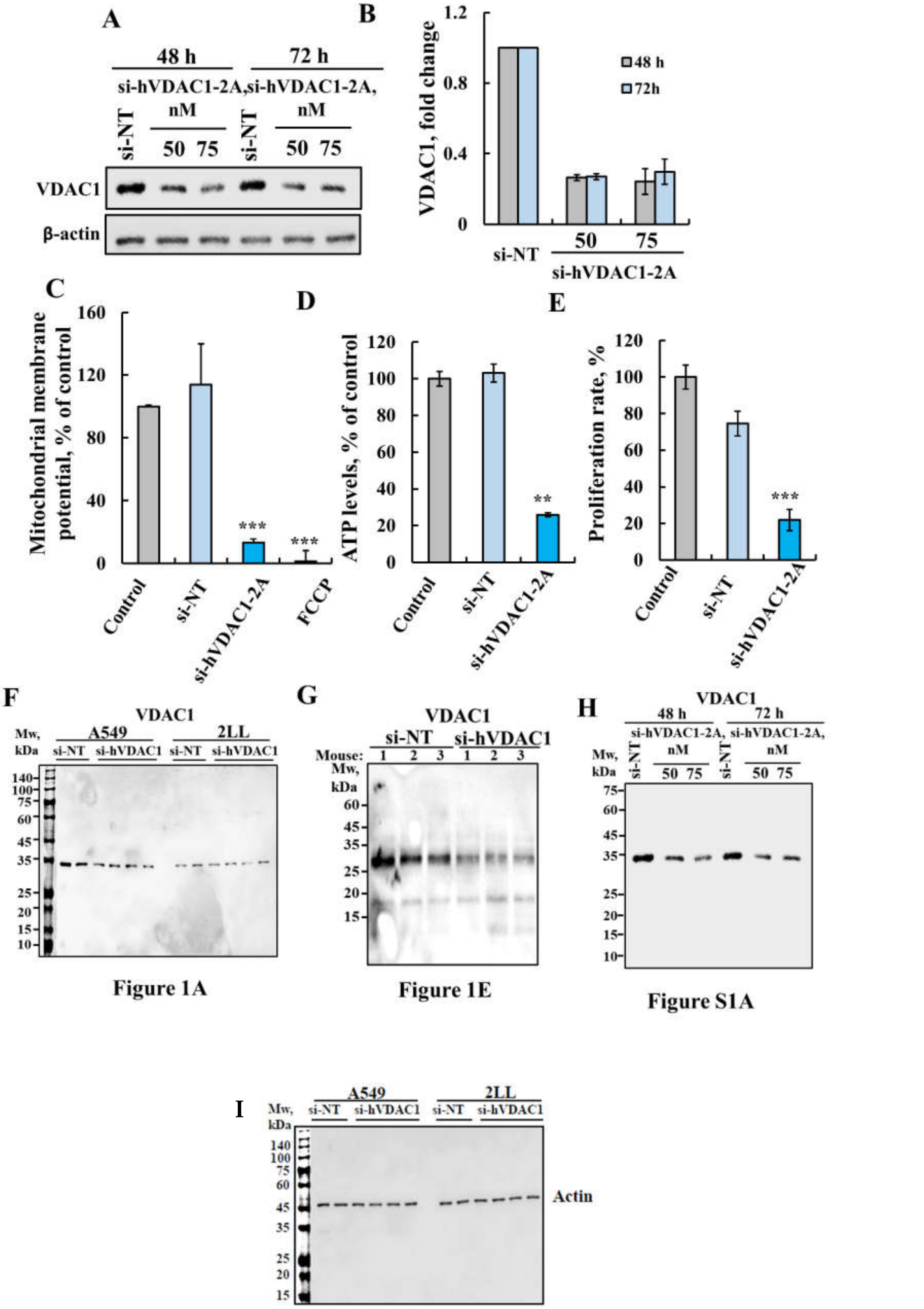


**Figure S1.** si-hVDAC1 inhibited VDAC1 expression, cell growth and reduced energy production in A549 cells. A549 cells were treated for 48 or 72 h with si-NT or si-hVDAC1-2A (50 or 75 nM) and analyzed for VDAC1 levels by immunoblotting (**A**) with the relative expression levels (**B**), mitochondria membrane potential $\Delta\Psi$ (**C**) and ATP (**D**) levels were analyzed ($n = 3$). Cell proliferation was analyzed using a sulforhodamine B assay (**E**). RU = relative unit. FCCP (carbonyl cyanide-4-

(trifluoromethoxy)phenylhydrazone), (25 µM) served as control for decreasing ΔΨ levels. The results are the mean ** $P \leq .01$; *** $P \leq 0.001$. Uncropped Western Blot of Fig. 1A (**F**), Fig. 1E (**G**) and Fig. S1A (**H**), β-actin bands (**I**).

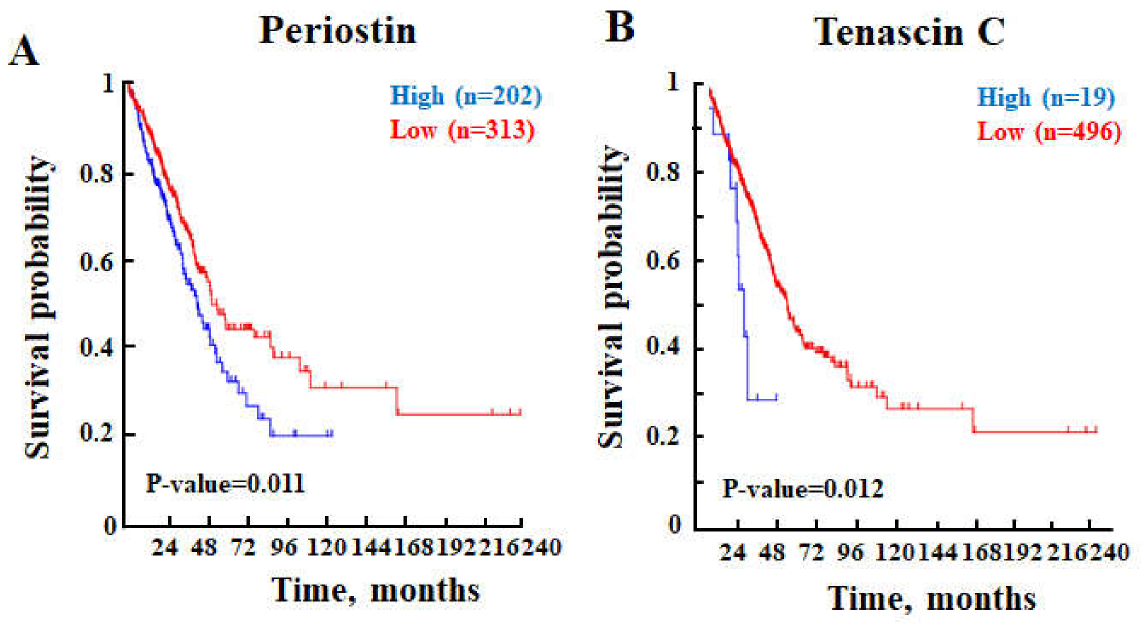


**Figure S2.** Decreased survival of patients with adenocarcinoma with high expression levels of periostin or tenascin C. (**A**,**B**) Kaplan–Meier analysis of overall survival curves of patients with adenocarcinoma based on low or high expression of periostin (**A**) or tenascin C (**B**). The *p*-values are indicated.

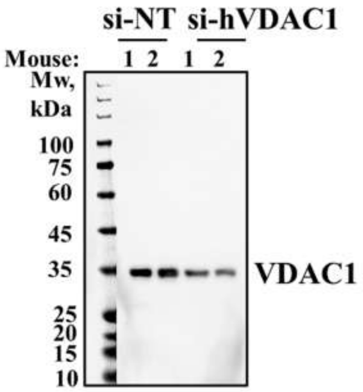


**Figure S3.** Uncropped Western Blot of Figure 9C.

**Table S3.** VDAC1 silencing in a A549 lung cancer-derived tumor alters the expression of mouse_ECM-related structural genes.

| No | Gene Name (Ensembl) | Fold change- si-hVDAC1/siNT (*P* value) | Proposed Function |
|---|---|---|---|
| | | **Collagen and collagen associated proteins** | |
| 1 | *Collagen, type XXVII, alpha 1 (Col27a1) (ENSMUSG00000045672)* | −1.93 (0.0766) | Plays a role during cartilage calcification and in the transition of cartilage to bone |

| | | | |
|---|---|---|---|
| 2 | *Collagen, type XVIII, alpha 1 (Col18a1) (ENSMUSG00000001435)* | −1.99 (0.0031) | Regulates ECM-dependent motility and morphogenesis of endothelial and non-endothelial cells. Inhibits endothelial cell proliferation and angiogenesis by binding to heparan sulfate proteoglycans |
| 3 | *Wingless-type MMTV integration site family, member 5A (Wnt5a) (ENSMUSG00000021994)* | −2 (0.0159) | Inhibits type II collagen expression in chondrocytes |
| 4 | *Cartilage associated protein (Crtap) (ENSMUSG00000032431)* | −2.18 (0.001) | Required for efficient 3-hydroxylation of fibrillar collagen prolyl residues |
| 5 | *Collagen, type XVI, alpha 1 (Col16a1) (ENSMUSG00000040690)* | −2.37 (5.66E−05) | Involved in mediating cell attachment and induces integrin-mediated cellular reactions, such as cell spreading and alterations in cell morphology |
| 6 | *Collagen, type IV, alpha 2 (Col4a2) (ENSMUSG00000031503)* | −2.57 (0.0223) | Major structural component of glomerular basement membranes, forming a 'chicken-wire' meshwork together with laminins, proteoglycans and entactin/nidogen |
| 7 | *Procollagen C-endopeptidase enhancer protein (Pcolce) (ENSMUSG00000029718)* | −2.58 (0.0279) | Binds to the C-terminal propeptide of type I procollagen and enhances its C-proteinase activity |
| 8 | *Collagen, type XIII, alpha 1 (Col13a1) (ENSMUSG00000058806)* | −2.58 (0.0322) | Involved in cell-matrix and cell-cell adhesion interactions required for normal development |
| 9 | *Collagen, type V, alpha 2 (Col5a2) (ENSMUSG00000026042)* | −2.75 (0.0215) | Group I collagen (fibrillar-forming collagen) |
| 10 | *Collagen, type IV, alpha 1 (Col4a1) (ENSMUSG00000031502)* | −2.76 (0.016) | Major structural component of glomerular basement membranes, forming a 'chicken-wire' meshwork together with laminins, proteoglycans and entactin/nidogen |
| 11 | *Collagen, type VI, alpha 1 (Col6a1) (ENSMUSG00000001119)* | −2.81 (0.0088) | Cell-binding protein |
| 12 | *Bone morphogenetic protein 1 (Bmp1) (ENSMUSG00000022098)* | −2.85 (0.0009) | Cleaves the C-terminal propeptides of procollagen I, II and III |
| 13 | *Collagen, type VI, alpha 2 (Col6a2) (ENSMUSG00000020241)* | −2.91 (0.0054) | Cell-binding protein |
| 14 | *Collagen, type V, alpha 3 (Col5a3) (ENSMUSG00000004098)* | −2.96 (0.0035) | Cell-matrix adhesion protein binds to DNA, heparan sulfate, thrombospondin, heparin, and insulin |
| 15 | *Collagen, type XXIV, alpha 1 (Col24a1) (ENSMUSG00000028197)* | −3.01 (0.0114) | ECM structural component |
| 16 | *Biglycan (Bgn) (ENSMUSG00000031375)* | −3.03 (0.0081) | May be involved in collagen fiber assembly |
| 17 | *Collagen, type I, alpha 1 (Col1a1) (ENSMUSG00000001506)* | −3.15 (0.0149) | Group I collagen (fibrillar-forming collagen) |
| 18 | *Collagen, type V, alpha 1 (Col5a1) (ENSMUSG00000026837)* | −3.2 (0.0076) | Group I collagen (fibrillar-forming collagen). |
| 19 | *Collagen, type XXIII, alpha 1 (Col23a1) (ENSMUSG00000063564)* | −3.29 (0.0013) | Collagen biosynthesis and modifying enzymes. Collagen degradation |
| 20 | *Collagen, type I, alpha 2 (Col1a2) (ENSMUSG00000029661)* | −3.3 (0.012) | Group I collagen (fibrillar-forming collagen). |
| 21 | *Collagen, type VI, alpha 3 (Col6a3) (ENSMUSG00000048126)* | −3.34 (0.003) | Cell-binding protein. ECM organization |
| 22 | *Collagen, type VIII, alpha 1 (Col8a1) (ENSMUSG00000068196)* | −3.44 (0.0248) | Necessary for migration and proliferation of vascular smooth muscle cells and thus for maintaining vessel wall integrity and structure in atherogenesis |
| 23 | *Collagen, type III, alpha 1 (Col3a1) (ENSMUSG00000026043)* | −3.49 (0.0045) | Occurs in most soft connective tissues along with type I collagen |
| 24 | *Collagen, type XII, alpha 1 (Col12a1) (ENSMUSG00000032332)* | −3.7 (0.0096) | Interacts with type I collagen-containing fibrils |
| 25 | *Collagen triple helix repeat-containing 1 (Cthrc1) (ENSMUSG00000054196)* | −3.75 (0.013) | Act as a negative regulator of collagen matrix deposition |
| 26 | *Collagen, type XIV, alpha 1 (Col14a1) (ENSMUSG00000022371)* | −3.98 (0.0003) | Plays an adhesive role by integrating collagen bundles, and may associated with the surface of interstitial collagen fibrils via COL1 |
| 27 | *Collagen, type VIII, alpha 2 (Col8a2) (ENSMUSG00000056174)* | −4.17 (0.0156) | Necessary for migration and proliferation of vascular smooth muscle cells |
| 28 | *Fibromodulin (Fmod) (ENSMUSG00000041559)* | −4.32 (0.03) | Affects the rate of fibrils formation. May play a primary role in collagen fibrillogenesis |
| 29 | *Collagen, type XI, alpha 1 (Col11a1) (ENSMUSG00000027966)* | −4.39 (0.018) | May play an important role in fibrillogenesis by controlling lateral growth of collagen II fibrils |
| 30 | *Podocan (Podn) (ENSMUSG00000028600)* | −6.55 (2.04E−09) | Binds to type I collagen. Negatively regulates cell proliferation and migration in smooth muscle cells |

| | | | |
|---|---|---|---|
| | **Laminin and laminin-associated proteins** | | |
| 31 | *Laminin, beta 2 (Lamb2) (ENSMUSG00000052911)* | −1.86 (0.0021) | Binds to cells via a high affinity receptor |
| 32 | *Laminin, gamma 1 (Lamc1) (ENSMUSG00000026478)* | −2.06 (0.0081) | Binds to cells via a high affinity receptor |
| | *Anthrax toxin receptor 2 (ANTXR2) (ENSMUSG00000029338)* | −2.17 (0.002) | Required for cellular interactions with laminin and the ECM |
| 33 | *Laminin, alpha 2 (Lama2) (ENSMUSG00000019899)* | −2.34 (0.0938) | Binds to cells via a high affinity receptor |
| 34 | *Laminin B1 (Lamb1) (ENSMUSG00000002900)* | −2.45 (0.00046) | Binds to cells via a high affinity receptor |
| | *Thrombospondin 1 (Thbs1) (ENSMUSG00000040152)* | −2.53 (0.0054) | Adhesive glycoprotein that mediates cell-to-cell and cell-to-matrix interactions. Can bind to fibrinogen, fibronectin, laminin, type V collagen and integrins alpha-V/beta−1, alpha-V/beta−3 and alpha-IIb/beta−3 |
| 35 | *Laminin, alpha 4 (Lama4) (ENSMUSG00000019846)* | −2.72 (0.0139) | Binds to cells via a high affinity receptor |
| | *Slit homolog 2 (Drosophila) (Slit2) (ENSMUSG00000031558)* | −4.05 (0.016691) | Laminin−1 binding |
| 36 | *Lumican (Lum) (ENSMUSG00000036446)* | −4.2 (6.33E−08) | Laminin binding |
| | **Fibronectin** | | |
| 38 | *Periostin, osteoblast specific factor (Postn) (ENSMUSG00000027750)* | −2.51 (0.0105) | Induces cell attachment and spreading and plays a role in cell adhesion. Enhances incorporation of BMP1 in the fibronectin matrix of connective tissues, and subsequent proteolytic activation of lysyl oxidase |
| 39 | *Fibronectin 1 (Fn1) (ENSMUSG00000026193)* | −2.83 (0.0283) | Involved in cell adhesion, cell motility, opsonization, wound healing, and maintenance of cell shape and cell shape healing |
| 40 | *Fibulin 2 (Fbln2) (ENSMUSG00000064080)* | −3.01 (0.002) | Its binding to fibronectin and other ligands is calcium-dependent. May act as an adapter that mediates the interaction between FBN1 and ELN |
| 41 | *Epidermal growth factor-containing fibulin-like extracellular matrix protein 2 (Efemp2) (ENSMUSG00000024909)* | −3.22 (0.0016) | Belongs to the fibulin family involved in elastic fiber assembly |
| 42 | *Fibulin 1 (Fbln1) (ENSMUSG00000006369)* | −4.56 (4.28E−07) | Incorporated into fibronectin-containing matrix fibers. May play a role in cell adhesion and migration along protein fibers within the ECM |
| | **Nidogen** | | |
| 43 | *Nidogen 1 (Nid1) (ENSMUSG00000005397)* | −2.29 (0.049) | Sulfated glycoprotein widely distributed in basement membranes and tightly associated with laminin. Binds to collagen IV and perlecan. May function in cell-ECM interactions |
| 44 | *Nidogen 2 (Nid2) (ENSMUSG00000021806)* | −3.4 (0.0138) | Cell adhesion glycoprotein. Probably plays a role in cell-extracellular matrix interactions |
| | Fibrillin | | |
| 45 | *Fibrillin 1 (Fbn1) (ENSMUSG00000027204)* | −2.99 (0.016) | Structural component of the 10−12 nm diameter microfibrils of the ECM. Conveys both structural and regulatory properties to load-bearing connective tissues |
| 46 | *Fibrillin 2 (Fbn2) (ENSMUSG00000024598)* | −3.07 (0.057) | Fibrillin−2-containing microfibrils regulate the early process of elastic fiber assembly |
| | **Collagen synthesis-associated proteins** | | |
| 47 | *Arginase−1 (ARG1) (ENSMUSG00000019987)* | 4.55 (0.0098) | Key element of the urea cycle, converting L-arginine to urea and L-ornithine, which is further metabolized into the metabolites proline and polyamides that drive collagen synthesis |
| 48 | *Translocating chain-associated membrane protein 2 (TRAM2) (ENSMUSG00000041779)* | −1.8 (0.057) | Necessary for collagen type I synthesis |
| | **Heparan sulfate-associated genes** | | |
| 49 | *Heparan sulfate glucosamine 3-O-sulfotransferase 6 (HS3ST6) (ENSMUSG00000039628)* | 14.15 (0.058) | Utilizes 3′-phospho−5′-adenylyl sulfate to catalyze the transfer of a sulfo group to heparan sulfate |
| 50 | *Bifunctional heparan sulfate N-deacetylase/N-sulfotransferase 1 (NDST1) (ENSMUSG00000054008)* | −1.68 (0.003) | Participates in biosynthesis of heparan sulfate serving as L-selectin ligands, thereby playing a role in inflammatory response |
| 51 | *Xylosyltransferase 2 (XYLT2) (ENSMUSG00000020868)* | −1.7 (0.032) | Involved in formation of heparan sulfate and chondroitin sulfate proteoglycans |
| 52 | *Exostosin−2 (EXT2) (ENSMUSG00000027198)* | −1.7 (0.0008) | Glycosyltransferase required for biosynthesis of heparan sulfate |

| | | | |
|---|---|---|---|
| 53 | *Exostoses (Multiple)-like 3 (EXTL3) (ENSMUSG00000021978)* | −1.72 (0.0001) | Glycosyltransferase which regulates biosynthesis of heparan sulfate |
| 54 | *Glypican 1 (Gpc1) (ENSMUSG00000034220)* | −1.76 (0.0003) | Cell surface proteoglycan that bears heparan sulfate |
| 55 | *Glypican 4 (Gpc4) (ENSMUSG00000031119)* | −1.86 (0.0163) | Cell surface proteoglycan that bears heparan sulfate |
| 56 | *Heparan sulfate 2-O-sulfotransferase 1 (HS2ST1) (ENSMUSG00000040151)* | −1.98 (0.00001) | Catalyzes transfer of sulfate to the C2-position of selected hexuronic acid residues within maturing heparan sulfate |
| 57 | *Perlecan (heparan sulfate proteoglycan 2) (Hspg2) (ENSMUSG00000028763)* | −2.18 (0.007) | Integral component of basement membranes where it is responsible for the fixed negative electrostatic charge and is involved in charge-selective ultrafiltration |
| 58 | *Heparan sulfate glucosamine 3-O-sulfotransferase 1 (HS3ST1) (ENSMUSG00000051022)* | −2.27 (0.03) | Catalyzes the rate-limiting step in heparan sulfate biosynthesis |
| 59 | *Glypican 6 (Gpc6) (ENSMUSG00000058571)* | −2.38 (0.0471) | Cell surface proteoglycan that bears heparan sulfate. Putative cell surface co-receptor for growth factors, extracellular matrix proteins, proteases and anti-proteases |
| 60 | *Glypican 3 (Gpc3) (ENSMUSG00000055653)* | −2.68 (0.05482) | Cell surface proteoglycan that bears heparan sulfate |
| 61 | *Extracellular sulfatase Sulf–1 (SULF1) (ENSMUSG00000016918)* | −2.72 (0.03) | Diminishes heparan sulfate proteoglycan sulfation. Inhibits signaling by heparin-dependent growth factors. Diminishes proliferation |
| 62 | *Heparan sulfate glucosamine 3-O-sulfotransferase 6 (HS3ST6) (ENSMUSG00000039628)* | 14.15 (0.058) | Utilizes 3′-phospho–5′-adenylyl sulfate to catalyze the transfer of a sulfo group to heparan sulfate |
| 63 | *Bifunctional heparan sulfate N-deacetylase/N-sulfotransferase 1 (NDST1) (ENSMUSG00000054008)* | −1.68 (0.003) | Participates in biosynthesis of heparan sulfate serving as L-selectin ligands, thereby playing a role in inflammatory response |
| 64 | *Xylosyltransferase 2 (XYLT2) (ENSMUSG00000020868)* | −1.7 (0.032) | Involved in formation of heparan sulfate and chondroitin sulfate proteoglycans |
| 65 | *Exostosin−2 (EXT2) (ENSMUSG00000027198)* | −1.7 (0.0008) | Glycosyltransferase required for biosynthesis of heparan sulfate |
| 66 | *Exostoses (Multiple)-like 3 (EXTL3) (ENSMUSG00000021978)* | −1.72 (0.0001) | Glycosyltransferase which regulates biosynthesis of heparan sulfate |
| 67 | *Glypican 1 (Gpc1) (ENSMUSG00000034220)* | −1.76 (0.0003) | Cell surface proteoglycan that bears heparan sulfate |
| 68 | *Glypican 4 (Gpc4) (ENSMUSG00000031119)* | −1.86 (0.0163) | Cell surface proteoglycan that bears heparan sulfate |
| 69 | *Heparan sulfate 2-O-sulfotransferase 1 (HS2ST1) (ENSMUSG00000040151)* | −1.98 (0.00001) | Catalyzes transfer of sulfate to the C2-position of selected hexuronic acid residues within maturing heparan sulfate |
| 70 | *Perlecan (heparan sulfate proteoglycan 2) (Hspg2) (ENSMUSG00000028763)* | −2.18 (0.007) | Integral component of basement membranes where it is responsible for the fixed negative electrostatic charge and is involved in charge-selective ultrafiltration |
| 71 | *Heparan sulfate glucosamine 3-O-sulfotransferase 1 (HS3ST1) (ENSMUSG00000051022)* | −2.27 (0.03) | Catalyzes the rate-limiting step in heparan sulfate biosynthesis |
| 72 | *Glypican 6 (Gpc6) (ENSMUSG00000058571)* | −2.38 (0.0471) | Cell surface proteoglycan that bears heparan sulfate. Putative cell surface co-receptor for growth factors, extracellular matrix proteins, proteases and anti-proteases |
| 73 | *Glypican 3 (Gpc3) (ENSMUSG00000055653)* | −2.68 (0.05482) | Cell surface proteoglycan that bears heparan sulfate |

Selected mouse genes from the down-regulated and up-regulated groups associated with ECM structure. The proposed function indicated. For each gene, the gene symbol and name, fold change in expression and *p*-value are indicated. Negative numbers reflect down-regulation.

**Table S4.** VDAC1 silencing in A549 lung cancer-derived tumor alters the expression of mouse ECM-organizing genes.

| No | Gene Name (Ensembl) | Fold change- si-hVDAC1/si-NT (*P* value) | Proposed Function |
|---|---|---|---|
| | | **ECM Organization** | |
| 1 | *Procollagen-lysine,2-oxoglutarate 5-dioxygenase 3 (PLOD3) (ENSMUSG00000004846)* | −1.6 (0.046) | Forms hydroxylysine residues in Xaa-Lys-Gly sequences in collagens that serve as sites of attachment for carbohydrate units and which are essential for the stability of intermolecular collagen cross-links |
| 2 | *Decorin (Dcn) (ENSMUSG00000019929)* | −1.9 (0.031697) | ECM organization. |

| | | | |
|---|---|---|---|
| 3 | *Cysteine-rich secretory protein LCCL domain-containing 2 (Crispld2) (ENSMUSG00000031825)* | −2.03 (0.0241) | Promotes matrix assembly |
| 4 | *von Willebrand factor A domain-containing 1 (Vwa1) (ENSMUSG00000042116)* | −2.03 (0.037102) | Promotes matrix assembly |
| 5 | *Alpha−1-syntrophin (SNTA1) (ENSMUSG00000027488)* | −2.12 (0.0007) | Adapter protein that binds to and probably organizes the subcellular localization of a variety of membrane proteins. May link various receptors to the actin cytoskeleton and the ECM via the dystrophin glycoprotein complex |
| 6 | *Tenascin C (Tnc) (ENSMUSG00000028364)* | −2.41 (0.0221) | Extracellular matrix protein |
| 7 | *Layilin (LAYN) (ENSMUSG00000060594)* | −2.44 (0.058) | Receptor for hyaluronate |
| 8 | *Olfactomedin-like 2B (Olfml2b) (ENSMUSG00000038463)* | −2.53 (0.0368) | Extracellular matrix binding |
| 9 | *Prolyl 3-hydroxylase 1 (P3h1) (ENSMUSG00000028641)* | −2.71 (0.005153) | Catalyzes the post-translational formation of 3-hydroxyproline in Xaa-Pro-Gly sequences in collagens, especially types IV and V |
| 10 | *Matrix-remodeling-associated protein 7 (MXRA7) (ENSMUSG00000020814)* | −2.76 (0.009) | ECM protein |
| 11 | *Procollagen-lysine,2-oxoglutarate 5-dioxygenase 2 (PLOD2) (ENSMUSG00000032374)* | −2.8 (0.016) | Forms hydroxylysine residues in Xaa-Lys-Gly sequences in collagens essential for the stability of intermolecular collagen cross-links |
| 12 | *Discoidin domain-containing receptor 2 (DDR2) (ENSMUSG00000026674)* | −2.8 (0.0002) | Tyrosine kinase that functions as cell surface receptor for fibrillar collagen and regulates remodeling of the ECM by upregulating the collagenases MMP1, MMP2 and MMP13 |
| 13 | *Peroxidasin (Pxdn) (ENSMUSG00000020674)* | −3.04 (0.000009) | Plays a role in ECM formation |
| 14 | *Serine (or cysteine) peptidase inhibitor, clade E, member 2 (Serpine2) (ENSMUSG00000026249)* | −3.08 (0.0168) | Serine protease inhibitor. Promotes neurite extension by inhibiting thrombin. Binds heparin |
| 15 | *Versican (Vcan) (ENSMUSG00000021614)* | −3.31 (0.0008) | May play a role in intercellular signaling and in connecting cells with the ECM |
| 16 | *Proline arginine-rich end leucine-rich repeat (Prelp) (ENSMUSG00000041577)* | −3.36 (0.0002) | May anchor basement membranes to underlying connective tissue |
| 17 | *Olfactomedin-like 2A (Olfml2a) (ENSMUSG00000046618)* | −3.56 (0.0016) | ECM organization |
| 18 | *SPARC-related modular calcium-binding 1 (Smoc1) (ENSMUSG00000021136)* | −3.82 (0.006) | ECM organization |
| 19 | *Fras1-related extracellular matrix protein 1 (Frem1) (ENSMUSG00000059049)* | −3.83 (0.0324) | Plays a role in epidermal differentiation and required for epidermal adhesion during embryonic development |
| 20 | *Tolloid-like protein 1 (TLL1) (ENSMUSG00000053626)* | −3.88 (0.002) | Protease processing of procollagen Cpropeptides, such as chordin, pro-biglycan and pro-lysyl oxidase. ECM disassembly |
| 21 | *ABI gene family, member 3 (NESH)-binding protein (Abi3bp) (ENSMUSG00000035258)* | −4.18 (0.00000000003) | ECM organization |
| 22 | *Homeobox protein Mohawk (MKX) (ENSMUSG00000061013)* | −5.16 (0.027) | Collagen fibril organization and morphogenetic regulator of cell adhesion |
| **Other ECM organization-associated genes** | | | |
| 23 | *F-box/LRR-repeat protein 14 (FBXL14) (ENSMUSG00000030019)* | 1.53 (0.047) | Stimulates SNAI1 degradation |
| 24 | *Zinc finger E-box-binding homeobox 1 (ZEB1) (ENSMUSG00000024238)* | −1.91 (0.056) | Acts as a transcriptional regulator in mesenchymal cells. Represses E-cadherin promoter. Induces epithelial-mesenchymal transition |
| 25 | *Kielin/chordin-like protein (KCP) (ENSMUSG00000059022)* | −2.07 (0.06) | Enhances bone morphogenetic protein signaling in a paracrine manner. Inhibits both the activin-A and TGFB1-mediated signaling pathways |
| 26 | *Protein TMEPAI (PMEPA1) (ENSMUSG00000038400)* | −2.1 (0.006) | Functions as a negative regulator of TGF-beta signaling |
| 27 | *Neogenin (NEO1) (ENSMUSG00000032340)* | −2.52 (0.0017) | Negative regulator of BMP signaling pathway |
| 28 | *Vasorin (VASN) (ENSMUSG00000039646)* | −2.55 (0.004) | Inhibitor of TGF-beta signaling. |
| 29 | *Zinc finger protein SNAI1 (SNAI1) (ENSMUSG00000042821)* | −3.07 (0.01) | Transcriptional regulator in mesenchymal cells. Represses E-cadherin promoter. Induces epithelial-mesenchymal transition |

| | | | |
|---|---|---|---|
| 30 | *Twist-related protein 1 (TWIST1) (ENSMUSG00000035799)* | −3.19 (0.001) | Transcriptional regulator in mesenchymal cells. Represses E-cadherin promoter. Induces an epithelial-mesenchymal transition. Homodimers induce expression of FGFR2 and POSTN while heterodimers repress FGFR2 and POSTN expression and induce THBS1 expression |
| 31 | *Prolyl 4-hydroxylase subunit alpha−3 ( P4HA3) (ENSMUSG00000051048)* | −3.68 (0.005) | Catalyzes the post-translational formation of 4-hydroxyproline in Xaa-Pro-Gly sequences in collagens and other proteins |
| 32 | *Bone morphogenetic protein receptor type−1B (BMPR1B) (ENSMUSG00000052430)* | −4.12 (0.015) | BMP signaling pathway |

Selected mouse genes from the down-regulated and up-regulated groups associated with ECM organization with their proposed function indicated. For each gene, the gene symbol and name, fold change in expression and p-value are indicated. Negative numbers reflect down-regulation.

**Table S5.** VDAC1 silencing in A549 lung cancer-derived tumour alters the expression of mouse ECM-remodelling-associated genes.

| **No** | **Gene Name (Ensembl)** | **Fold change-sihVDAC1/si-NT (P value)** | **Proposed Function** |
|---|---|---|---|
| | | **Metallopeptidases** | |
| 1 | *Macrophage metalloelastase (MMP12) (ENSMUSG00000049723)* | 4.4 (0.010) | May be involved in tissue injury and remodeling. Has significant elastolytic activity |
| 2 | *Tissue inhibitor of metalloproteinase 1 (Timp1) (ENSMUSG00000001131)* | −2.11 (0.1063) | Metalloproteinase inhibitor |
| 3 | *matrix metallopeptidase 17 (Mmp17) (ENSMUSG00000029436)* | −2.3 (0.1049) | Endopeptidase that degrades various components of the ECM, such as fibrin. Involved in the activation of membrane-bound precursors of growth factors or inflammatory mediators |
| 4 | *Tissue inhibitor of metalloproteinase 2  (Timp2) (ENSMUSG00000017466)* | −2.38 (0.0049) | Complexes with metalloproteinases (such as collagenases) and irreversibly inactivates them. |
| 5 | *Reversion-inducing cysteine-rich protein with Kazal motifs (RECK) (ENSMUSG00000028476)* | −2.51 (0.008) | Negatively regulates MMP−9. Appears to also regulate MMP−2 and MT1-MMP, which are involved in cancer progression |
| 6 | *Matrix metallopeptidase 14 (membrane-inserted) (Mmp14) (ENSMUSG00000000957)* | −2.55 (0.0117) | Endopeptidase that degrades various components of the extracellular matrix, such as collagen. Activates progelatinase A. Essential for pericellular collagenolysis |
| 7 | *Matrix metallopeptidase 23 (Mmp23) (ENSMUSG00000029061)* | −2.57 (0.0566) | Metalloproteinase. Regulates the surface expression of some potassium channels by retaining them in the ER |
| 8 | *Matrix metallopeptidase 11 (Mmp11) (ENSMUSG00000000901)* | −2.8 (0.0009) | Extracellular matrix disassembly, collagen catabolic process. May play an important role in the progression of epithelial malignancies |
| 9 | *Discoidin domain-containing receptor 2 (DDR2) (ENSMUSG00000026674)* | −2.8 (0.0002) | Tyrosine kinase that functions as cell surface receptor for fibrillar collagen and regulates remodeling of the extracellular matrix, by up-regulation of the collagenases MMP1, MMP2 and MMP13 |
| 10 | *Matrix metallopeptidase 13 (Mmp13) (ENSMUSG00000050578)* | −3.17 (0.0178) | Plays a role in the degradation of extracellular matrix proteins including fibrillar collagen, fibronectin, TNC and ACAN |
| 11 | *Matrix metallopeptidase 3 (Mmp3) (ENSMUSG00000043613)* | −3.46 (0.0078) | Can degrade fibronectin, laminin, gelatins of type I, III, IV, and V; collagens III, IV, X, and IX, and cartilage proteoglycans |
| 12 | *Matrix metallopeptidase 27 (Mmp27) (ENSMUSG00000070323)* | −3.48 (0.1157) | Metalloendopeptidase activity  degrade protein components of the extracellular matrix such as fibronectin, laminin, gelatins and/or collagens |
| 13 | *Matrix metallopeptidase 9 (Mmp9) (ENSMUSG00000017737)* | −4.25 (0.0531) | Cleaves type IV and type V collagen into large C-terminal three quarter fragments and shorter N-terminal one quarter fragments |
| 14 | *Matrix metallopeptidase 2 (Mmp2) (ENSMUSG00000031740)* | −4.27 (3.50E−05) | Cleavage of gelatin type I and collagen types IV, V, VII, X. Cleaves the collagen-like sequence Pro-Gln-Gly-|-Ile-Ala-Gly-Gln |
| | | **ADAM and ADAMTS** | |
| 15 | *A disintegrin and metalloproteinase domain-containing protein 9  (ADAM9) (ENSMUSG00000031555)* | −1.69 (0.031) | Cleaves and releases molecules involved in tumorigenesis and angiogenesis, such as TEK, KDR, EPHB4, CD40, VCAM1 and CDH5. May mediate cell-cell, cell-matrix interactions and regulate the motility of cells via interactions with integrins |

| | | | |
|---|---|---|---|
| 16 | *A disintegrin-like and metallopeptidase (reprolysin type) with thrombospondin type 1 motif, 9 (Adamts9) (ENSMUSG00000030022)* | −1.85 (0.0535) | Extracellular matrix organization, Cleaves the large aggregating proteoglycans, aggrecan and versican |
| 17 | *A disintegrin-like and metallopeptidase (reprolysin type) with thrombospondin type 1 motif, 1 (Adamts1) (ENSMUSG00000022893)* | −1.9 (0.038) | Cleaves aggrecan, a cartilage proteoglycan, Has angiogenic inhibitor activity. Active metalloprotease, which may be associated with various inflammatory processes as well as development of cancer cachexia |
| 18 | *GDP-fucose protein O-fucosyltransferase 2 (POFUT2) (ENSMUSG00000020260)* | −1.96 (0.006) | Required for the proper secretion of ADAMTS family members such as ADAMSL1 and ADAMST13. O-fucosylation of TSRs, also required for restricting EMT |
| 19 | *Disintegrin and metalloproteinase domain-containing protein 23 (ADAM23) (ENSMUSG00000025964)* | −2.25 (0.065) | May play a role in cell-cell and cell-matrix interactions. Non-catalytic metalloprotease-like protein |
| 20 | *A disintegrin-like and metallopeptidase (reprolysin type) with thrombospondin type 1 motif, 5 (aggrecanase−2) (Adamts5) (ENSMUSG00000022894)* | −2.45 (0.0184) | ECM-degrading enzyme that shows proteolytic activity toward the hyalectan group of chondroitin sulfate proteoglycans, including aggrecan, versican, brevican and neurocan. |
| 21 | *A disintegrin-like and metallopeptidase (reprolysin type) with thrombospondin type 1 motif, 7 (Adamts7) (ENSMUSG00000032363)* | −2.51 (0.04817) | Metalloprotease that may play a role in the degradation of COMP |
| 22 | *A disintegrin-like and metallopeptidase (reprolysin type) with thrombospondin type 1 motif, 4 (Adamts4) (ENSMUSG00000006403)* | −2.97 (0.0322) | Cleaves aggrecan, a cartilage proteoglycan |
| 23 | *A disintegrin-like and metallopeptidase (reprolysin type) with thrombospondin type 1 motif, 2 (Adamts2) (ENSMUSG00000036545)* | −2.97 (0.008) | Cleaves the propeptides of type I and II collagen prior to fibril assembly. Does not act on type III collagen |
| 24 | *A disintegrin-like and metallopeptidase (reprolysin type) with thrombospondin type 1 motif, 15 (Adamts15) (ENSMUSG00000033453)* | −3.45 (0.0042) | Metalloendopeptidase activity |
| 25 | *Disintegrin and metalloproteinase domain-containing protein 12 (ADAM12) (ENSMUSG00000054555)* | −3.7 (0.006) | Metallopeptidase activity and ECM organization |
| 26 | *Disintegrin and metalloproteinase domain-containing protein 22 (ADAM22) (ENSMUSG00000040537)* | −4.67 (0.032) | Probable ligand for integrin in the brain. Non-catalytic metalloprotease-like protein. Involved in regulation of cell adhesion and spreading and in inhibition of cell proliferation. Neuronal receptor for LGI1 |
| 27 | *A disintegrin-like and metallopeptidase (reprolysin type) with thrombospondin type 1 motif, 12 (Adamts12) (ENSMUSG00000047497)* | −5.09 (0.0055) | Metalloprotease that plays a role in the degradation of Cartilage oligomeric matrix protein (COMP) (By similarity). Cleaves also alpha−2 macroglobulin and aggregan. Has anti-tumorigenic properties |
| 28 | *A disintegrin-like and metallopeptidase (reprolysin type) with thrombospondin type 1 motif, 3 (Adamtsl3) (ENSMUSG00000070469)* | −5.58 (0.0247) | Metalloendopeptidase activity |
| 29 | *A disintegrin-like and metallopeptidase (reprolysin type) with thrombospondin type 1 motif, 16 (Adamts16) (ENSMUSG00000049538)* | −8.38 (0.0052) | Metalloendopeptidase activity, tight interaction with ECM |
| **Other metalloproteinases and regulator proteins** | | | |
| 30 | *Kallikrein−8 (KLK8) (ENSMUSG00000064023)* | 6.41 (0.019) | Serine protease capable of degrading a number of proteins, such as casein, fibrinogen, kininogen, fibronectin and collagen type IV |
| 31 | *Prolyl endopeptidase FAP (FAP) (ENSMUSG00000000392)* | −3.5 (0.002) | Cell surface glycoprotein serine protease that participates in ECM degradation and involved in many cellular processes including tissue remodeling |
| 32 | *Tetraspanin−12 (TSPAN12) (ENSMUSG00000029669)* | −3.78 (0.0018) | Regulator of membrane proteinases such as ADAM10 and MMP14/MT1-MMP. Activates ADAM10-dependent cleavage activity of amyloid precursor protein (APP). Activates MMP14/MT1-MMP-dependent cleavage activity |
| 33 | *Pappalysin−1 (PAPPA) (ENSMUSG00000028370)* | −4.32 (0.0001) | Metalloproteinase which specifically cleaves IGFBP−4 and IGFBP−5, resulting in release of bound IGF. Cleavage of IGFBP−4 is dramatically enhanced by the presence of IGF, whereas cleavage of IGFBP−5 is slightly inhibited by the presence of IGF |
| 34 | *Cathepsin K (CTSK) (ENSMUSG00000028111)* | −4.97 (0.002) | Plays an important role in ECM degradation |
| **Lysyl oxidase** | | | |

| | | | |
|---|---|---|---|
| 35 | *Lysyl oxidase-like 2 (Loxl2) (ENSMUSG00000034205)* | −2.4 (0.0382) | When secreted in ECM, promotes cross-linking of ECM proteins by mediating oxidative deamination of peptidyl lysine residues in precursors to fibrous collagen and elastin |
| 36 | *Lysyl oxidase homolog 4 (LoxL4) (ENSMUSG00000025185)* | −2.51 (0.074) | May modulate formation of a collagenous ECM |
| 37 | *Lysyl oxidase (Lox) (ENSMUSG00000024529)* | −2.6 (0.0484) | Responsible for the post-translational oxidative deamination of peptidyl lysine residues in precursors to fibrous collagen and elastin |
| 38 | *Lysyl oxidase-like 1 (Loxl1) (ENSMUSG00000032334)* | −3.4 (0.0041) | Active on elastin and collagen |

Selected mouse genes from the down-regulated and up-regulated groups associated with ECM remodelling. The proposed function indicated. For each gene, the gene symbol and name, fold change in expression and *p*-value are indicated. Negative numbers reflect down-regulation.

**Table S6.** VDAC1 silencing in a A549 lung cancer-derived tumor alters the expression of mouse genes for intercellular-interacting proteins.

| No | Gene Name ( Ensembl) | Fold change si-hVDAC1/si-NT (P value) | Proposed Function |
|---|---|---|---|
| | | **Intercellular interaction** | |
| 1 | *Leucine-rich repeat transmembrane protein FLRT3 (FLRT3) (ENSMUSG00000051379)* | 9.05 (0.020) | Plays a role in cell-cell adhesion via interaction with ADGRL3. Plays a role in fibroblast growth factor-mediated signaling cascades |
| 2 | *L1 cell adhesion molecule (L1CAM) (ENSMUSG00000031391)* | 3.43 (0.039) | Neuronal cell adhesion molecule with a strong implication in cell migration |
| 3 | *Basal cell adhesion molecule (BCAM) (ENSMUSG00000002980)* | 3.41 (0.023) | Laminin alpha−5 receptor. May mediate intracellular signaling |
| 4 | *Tensin 1 (TNS1) (ENSMUSG00000055322)* | −1.69 (0.021) | Involved in fibrillar adhesion formation. cell-substrate junction assembly |
| 5 | *Lectin, galactose binding, soluble 1 (Lgals1) (ENSMUSG00000068220)* | −1.71 (0.056681) | Lectin that binds beta-galactoside and a wide array of complex carbohydrates |
| 6 | *Liprin-beta−1 (PPFIBP1) (ENSMUSG00000016487)* | −1.84 (0.0002) | Cell adhesion protein |
| 7 | *Protocadherin gamma subfamily C, 4 (PCDHGC4) (ENSMUSG00000023036)* | −1.88 (0.0007) | Potential calcium-dependent cell-adhesion protein |
| 8 | *Pseudopodium-enriched atypical kinase 1 (PEAK1) (ENSMUSG00000074305)* | −1.92 (0.017) | Tyrosine kinase that may play a role in cell spreading and migration on fibronectin |
| 9 | *Vinculin (VCL) (ENSMUSG00000021823)* | −2.01 (0.007) | Actin filament (F-actin)-binding protein involved in cell-matrix adhesion and cell-cell adhesion |
| 10 | *SPARC related modular calcium binding 2 (Smoc2) (ENSMUSG00000023886)* | −2.36 (0.0116) | Promotes matrix assembly and cell adhesiveness |
| 11 | *Extracellular matrix protein 2, female organ and adipocyte specific (Ecm2) (ENSMUSG00000043631)* | −2.4 (0.0513) | Promotes matrix assembly and cell adhesiveness |
| 12 | *Spondin 1, (f-spondin) extracellular matrix protein (Spon1) (ENSMUSG00000038156)* | −2.62 (0.046) | Cell adhesion protein |
| 13 | *EGF-like, fibronectin type III and laminin G domains (Egflam) (ENSMUSG00000042961)* | −2.63 (0.0599) | Promotes matrix assembly and cell adhesiveness |
| 14 | *Cadherin−2 (CDH2) (NCAD) (ENSMUSG00000024304)* | −2.71 (0.075) | Associated with EMT |
| 15 | *Microfibrillar associated protein 5 (Mfap5) (ENSMUSG00000030116)* | −2.71 (0.0266) | Component of the elastin-associated microfibrils |
| 16 | *Fibulin 5 (Fbln5) (ENSMUSG00000021186)* | −2.72 (0.0014) | Essential for elastic fiber formation. Involved in assembly of continuous elastin polymer. Promotes interaction of microfibrils and ELN |
| 17 | *Carboxypeptidase X 2 (M14 family) (Cpxm2) (ENSMUSG00000030862)* | −2.86 (0.0011) | May be involved in cell-cell interactions |
| 18 | *Microfibrillar-associated protein 2 (Mfap2) (ENSMUSG00000060572)* | −2.88 (0.0016) | Component of the elastin-associated microfibrils |

| | | | |
|---|---|---|---|
| 19 | *SPARC-like 1 (Sparcl1) (ENSMUSG00000029309)* | −2.92 (0.01481) | Interacts with the ECM to create intermediate states of cell adhesion |
| 20 | *Secreted acidic cysteine rich glycoprotein (Sparc) (ENSMUSG00000018593)* | −3.05 (0.01485) | Appears to regulate cell growth through interactions with the ECM and cytokines. Binds calcium and copper, several types of collagen, albumin, thrombospondin, PDGF and cell membranes |
| 21 | *CD93 antigen (Cd93) (ENSMUSG00000027435)* | −3.07 (0.0082) | Interacts with soluble defense collagens. May play a role in intercellular adhesion |
| 22 | *Neural cell adhesion molecule 1 (NCAM1) (ENSMUSG00000039542)* | −3.09 (0.004) | Cell adhesion |
| 23 | *Coiled-coil domain containing 80 (Ccdc80) (ENSMUSG00000022665)* | −3.21 (0.00016) | Promotes cell adhesion and matrix assembly |
| 24 | *Thrombospondin 2 (Thbs2) (ENSMUSG00000023885)* | −3.24 (0.0078) | Adhesive glycoprotein that mediates cell-to-cell and cell-to-matrix interactions. Ligand for CD36 mediating antiangiogenic properties |
| 25 | *Cadherin−11 (CDH11) (ENSMUSG00000031673)* | −3.33 (0.006) | Adhesion protein |
| 26 | *Microfibrillar-associated protein 4 (Mfap4) (ENSMUSG00000042436)* | −3.42 (0.0027) | Could be involved in calcium-dependent cell adhesion or intercellular interactions |
| 27 | *Cartilage intermediate layer protein, nucleotide pyrophosphohydrolase (Cilp) (ENSMUSG00000042254)* | −3.43 (3.50E−05) | Has the ability to suppress IGF1-induced proliferation and sulfated proteoglycan synthesis. Inhibits ligand-induced IGF1R autophosphorylation |
| 28 | *Leucine-rich repeat-containing protein 15 (LRRC15) (ENSMUSG00000052316)* | −3.43 (0.0014) | Collagen binding, fibronectin binding, laminin binding |
| 29 | *Leucine-rich repeat and fibronectin type-III domain-containing protein 3 (LRFN3) (ENSMUSG00000036957)* | −3.49 (0.012) | Cell adhesion molecule that mediates homophilic cell-cell adhesion in a $Ca^{2+}$-independent manner |
| 30 | *Reelin (Reln) (ENSMUSG00000042453)* | −3.58 (0.0506) | ECM serine protease. Enzymatic activity is important for modulation of cell adhesion |
| 31 | *Thrombospondin−3 (THBS3) (ENSMUSG00000028047)* | −3.61 (0.0012) | Adhesive glycoprotein that mediates cell-to-cell and cell-to-matrix interactions. Can bind to fibrinogen, fibronectin, laminin and type V collagen |
| 32 | *Scavenger receptor cysteine rich family, 5 domains (Ssc5d) (ENSMUSG00000035279)* | −4.94 (1.15E−06) | Binds to ECM proteins |
| 33 | *Protocadherin 17 (PCDH17) (ENSMUSG00000035566)* | −5.85 (0.0095) | Homophilic cell adhesion via plasma membrane adhesion molecules |
| 34 | *Thrombospondin 4 (Thbs4) (ENSMUSG00000021702)* | −6.48 (0.0561) | Adhesive glycoprotein that mediates cell-to-cell and cell-to-matrix interactions. Can bind to fibrinogen, fibronectin, laminin and type V collagen |
| 35 | *Cartilage oligomeric matrix protein (Comp) (ENSMUSG00000031849)* | −6.71 (0.000247) | Interaction with ECM proteins, such as the collagens and fibronectin |
| 36 | *Cadherin−10 (Cdh10) (ENSMUSG00000022321)* | −20.43 (0.002) | Homophilic cell adhesion via plasma membrane adhesion molecules |
| | | **Integrin and growth factors** | |
| 37 | Integrin beta−4 (ITGB4) (ENSMUSG00000020758) | 3.14 (0.093) | Integrin alpha−6/beta−4 is a receptor for laminin. It plays a critical structural role in the hemidesmosome of epithelial cells. |
| 38 | Integrin beta (ITGB2) (ENSMUSG00000000290) | 3.1 (0.016) | It is a receptor for ICAM1, ICAM2, ICAM3 and ICAM4 |
| 39 | Integrin alpha-IIb (ITGA2B) (ENSMUSG00000034664) | 2.91 (0.044) | Integrin alpha-IIb/beta−3 is a receptor for fibronectin, fibrinogen, plasminogen, prothrombin, thrombospondin and vitronectin. |
| 40 | Integrin alpha-X (ITGAX) (ENSMUSG00000030789) | 2.41 (0.104) | Integrin alpha-X/beta−2 is a receptor for fibrinogen. It recognizes the sequence G-P-R in fibrinogen. It mediates cell-cell interaction during inflammatory responses. It is especially important in monocyte adhesion and chemotaxis. |
| 41 | Intercellular adhesion molecule 1 (ICAM1) (ENSMUSG00000037405) | 2.27 (0.017) | ICAM proteins are ligands for the leukocyte adhesion protein LFA−1 (integrin alpha-L/beta−2). |
| 42 | Golgi apparatus protein 1 (Glg1) (ENSMUSG00000003316) | −1.53 (0.012019) | Binds fibroblast growth factor. Binds E-selectin (cell-adhesion lectin on endothelial cells mediating the binding of neutrophils). |
| 43 | Integrin alpha-V (ITGAV) (ENSMUSG00000027087) | −1.6 (0.012) | The alpha-V (ITGAV) integrins are receptors for vitronectin, cytotactin, fibronectin, fibrinogen, laminin, matrix metalloproteinase−2, osteopontin, osteomodulin, prothrombin, thrombospondin and vWF. |
| 44 | LIM and senescent cell antigen-like-containing domain protein (LIMS1) (ENSMUSG00000019920) | −1.61 (0.016) | Adapter protein in a cytoplasmic complex linking beta-integrins to the actin cytoskeleton, bridges the complex to cell surface receptor tyrosine kinases and growth factor receptors. |

| | | | |
|---|---|---|---|
| 45 | Latent transforming growth factor beta binding protein 3 (Ltbp3) (ENSMUSG00000024940) | −1.61 (0.0088) | May be involved in the assembly, secretion and targeting of TGF-beta1 to sites at which it is stored and/or activated. May play critical roles in controlling and directing the activity of TGF-beta1. May have a structural role in the ECM. |
| 46 | Integrin beta (ITGB5) (ENSMUSG00000022817) | −1.83 (0.022) | Possible receptor for fibronectin. |
| 47 | Insulin-like growth factor I (IGF1) (ENSMUSG00000020053) | −1.91 (0.05) | The insulin-like growth factors, isolated from plasma, are structurally and functionally related to insulin but have a much higher growth-promoting activity. Acts as a ligand for IGF1R. Binds to integrins ITGAV:ITGB3 and ITGA6:ITGB4. Its binding to integrins and subsequent ternary complex formation with integrins and IGFR1 are essential for IGF1 signaling. |
| 48 | Endoglin (ENG) (ENSMUSG00000026814) | −1.92 (0.017) | Acts as TGF-beta co-receptor and is involved in the TGF-beta/BMP signaling cascade that ultimately leads to the activation of SMAD transcription factors |
| 49 | TGF-beta receptor type−2 (TGFBR2) (ENSMUSG00000032440) | −2.15 (0.012) | Transmembrane serine/threonine kinase forming with the TGF-beta type I serine/threonine kinase receptor. |
| 50 | Latent transforming growth factor beta binding protein 2 (Ltbp2) (ENSMUSG00000002020) | −2.24 (0.0678) | Play an integral structural role in elastic-fiber architectural organization and/or assembly. May be involved in the assembly, secretion and targeting of TGF- beta to sites at which it is stored and/or activated. Play critical roles in controlling and directing the activity of TGF-beta. May have a structural role in the ECM. |
| 51 | Latent transforming growth factor beta binding protein 1 (Ltbp1) (ENSMUSG00000001870) | −2.25 (0.0245) | Involved in the assembly, secretion and targeting of TGFB1 to sites at which it is stored and/or activated. May play critical roles in controlling and directing the activity of TGFB1. May have a structural role in the ECM. |
| 52 | Transforming growth factor beta 1 induced transcript 1 (Tgfb1i1) (ENSMUSG00000030782) | −2.55 (0.0053) | Links various intracellular signaling modules to plasma membrane receptors and regulates the Wnt and TGFB signaling pathways. |
| 53 | Transforming growth factor, beta 3 (Tgfb3) (ENSMUSG00000021253) | −2.65 (0.0244) | Regulates molecules involved in cellular adhesion and ECM formation |
| 54 | Transforming growth factor, beta 2 (Tgfb2) (ENSMUSG00000039239) | −3.18 (0.0001) | TGF-beta 2 has suppressive effects on interleukin−2 dependent T-cell growth. |
| 55 | Cell adhesion molecule 3 (CADM3) (ENSMUSG00000005338) | −3.19 (0.036) | Involved in the cell-cell adhesion. |
| 56 | Asporin (Aspn) (ENSMUSG00000021388) | −3.52 (0.0195) | Plays a role in osteoblast-driven collagen biomineralization activity. Critical regulator of TGF-beta in articular cartilage |
| 57 | Sushi, von Willebrand factor type A, EGF and pentraxin domain-containing protein 1 (SVEP1) (ENSMUSG00000028369) | −3.63 (0.0035) | Play a role in the cell adhesion process. |
| 58 | Epidermal growth factor-containing fibulin-like extracellular matrix protein 1 (Efemp1) (ENSMUSG00000020467) | −3.98 (0.0212) | Binds EGFR, the EGF receptor, inducing EGFR autophosphorylation and the activation of downstream signaling pathways. May play a role in cell adhesion and migration. |
| 59 | EGF-like repeats and discoidin I-like domains 3 (Edil3) (ENSMUSG00000034488) | −4.41 (0.0235) | Promotes adhesion of endothelial cells through interaction with the alpha-v/beta−3 integrin receptor. Inhibits formation of vascular-like structures. |
| 60 | Integrin beta-like protein 1 (ITGBL1) (ENSMUSG00000032925) | −4.74 (0.0038) | Cell adhesion |
| 61 | Nephroblastoma overexpressed gene (Nov) (ENSMUSG00000037362) | −5.12 (0.00197) | Playing a role in various cellular processes including proliferation, adhesion, migration, differentiation and survival. Acts by binding to integrins or membrane receptors such as NOTCH1. |
| 62 | Osteoglycin (Ogn) (ENSMUSG00000021390) | −5.48 (5.89E−06) | Induces bone formation in conjunction with TGF-beta−1 or TGF-beta−2. |
| 63 | Neurofascin (NFASC) (ENSMUSG00000026442) | −6.65 (0.00005) | Cell adhesion, ankyrin-binding protein which may be involved in neurite extension |
| 64 | Integrin binding sialoprotein (IBSP) (ENSMUSG00000029306) | −8.06 (0.0285) | Appears to form an integral part of the mineralized matrix. Probably important to cell-matrix interaction. |
| 65 | Tenascin N (TNN) (ENSMUSG00000026725) | −9.59 (9.61E−06) | Extracellular matrix protein that seems to be a ligand for ITGA8:ITGB1, ITGAV, ITGB1 and ITGA4, ITGB1 |

Selected mouse genes from the down-regulated and up-regulated groups associated with extracellular intercellular-interaction. The proposed function indicated. For each gene, the gene symbol and name, fold change in expression and p-value are indicated. Negative numbers reflect down-regulation.

**Table S7.** Alterations in the expression of angiogenesis-associated mouse genes in a si-hVDAC1-treated A549-derived xenograft.

| No | Gene Name (Ensembl) | Fold change si-hVDAC1/si-NT (P value) | Proposed Function |
|---|---|---|---|
| | | **Angiogenesis** | |
| 1 | *Interleukin−12 subunit alpha (IL12A) (ENSMUSG00000027776)* | 25.86 (0.036) | Angiostatic cytokine |
| 2 | *Ectonucleotide pyrophosphatase /phosphodiesterase family member 2 (ENPP2) (ENSMUSG00000022425)* | 16.23 (0.0003) | Acts as an angiogenic factor by stimulating migration of smooth muscle cells and microtubule formation |
| 3 | *Interferon gamma (IFNG) (ENSMUSG00000055170)* | 13.26 (0.0128) | Anti-angiogenic factor |
| 4 | *Zinc finger protein castor homolog 1 (CASZ1) (ENSMUSG00000028977)* | 10.85 (0.015) | Transcription factor involved in vascular assembly and morphogenesis through direct transcriptional regulation of EGFL7 |
| 5 | *Prospero homeobox protein 1 (PROX1) (ENSMUSG00000010175)* | 7.28 (0.0112) | Positive regulation of lymphangiogenesis |
| 6 | *C-X-C motif chemokine 10 (CXCL10) (ENSMUSG00000034855)* | 5.06 (0.0148) | Anti-angiogenic factor |
| 7 | *Receptor-type tyrosine-protein kinase FLT3 (FLT3) (ENSMUSG00000042817)* | 4.66 (0.0154) | Pro-angiogenic factor |
| 8 | *Protransforming growth factor alpha (TGFA) (ENSMUSG00000029999)* | 2.76 (0.0373) | Positive regulator of angiogenesis |
| 9 | *Integrin alpha-V (ITGAV) (ENSMUSG00000027087)* | −1.6 (0.012) | Positive regulator of angiogenesis |
| 10 | *Plexin domain-containing protein 2 (PLXDC2) (ENSMUSG00000026748)* | −1.81 (0.011) | Plays a role in tumor angiogenesis |
| 11 | *A disintegrin-like and metallopeptidase (reprolysin type) with thrombospondin type 1 motif, 1(Adamts1) (ENSMUSG00000022893)* | −1.9 (0.038) | Active metalloprotease which may be associated with various inflammatory processes, as well as development of cancer cachexia. Has angiogenic inhibitor activity |
| 12 | *Angiopoietin-related protein 2 (ANGPTL2) (ENSMUSG00000004105)* | −1.91 (0.02) | Pro-angiogenic factor |
| 13 | *collagen, type XVIII, alpha 1(Col18a1) (ENSMUSG00000001435)* | −1.99 (0.0031) | Potently inhibits endothelial cell proliferation and angiogenesis |
| 14 | *cysteine rich protein 61 (Cyr61) (ENSMUSG00000028195)* | −2.09 (0.0408) | Promotes cell proliferation, chemotaxis, angiogenesis and cell adhesion |
| 15 | *Von Willebrand factor (Vwf) (ENSMUSG00000001930)* | −2.14 (0.063104) | Pro-angiogenic factor |
| 16 | *perlecan (heparan sulfate proteoglycan 2) (Hspg2) (ENSMUSG00000028763)* | −2.18 (0.007) | Positive and negative regulator of angiogenesis |
| 17 | *Platelet factor 4 (PF4) (ENSMUSG00000029373)* | −2.22 (0.0359) | Negative regulator of angiogenesis |
| 18 | *Neuropilin−1 (NRP1) (ENSMUSG00000025810)* | −2.26 (0.0154) | Positive and negative regulator of angiogenesis |
| 19 | *multimerin 2(Mmrn2) (ENSMUSG00000041445)* | −2.27 (0.048801) | Negative regulator of angiogenesis |
| 20 | *connective tissue growth factor (Ctgf) (ENSMUSG00000019997)* | −2.28 (0.07594) | Involved in angiogenesis. Interacts with endothelial cells |
| 21 | *Metalloproteinase inhibitor 2 (TIMP2) (ENSMUSG00000017466)* | −2.38 (0.0049) | Positive regulation of angiogenesis in tumors |
| 22 | *Thrombospondin 1(Thbs1) (ENSMUSG00000040152)* | −2.53 (0.0054) | Positive and negative regulator of angiogenesis |
| 23 | *collagen, type IV, alpha 2 (Col4a2) (ENSMUSG00000031503)* | −2.57 (0.0223) | Negative regulator of angiogenesis |
| 24 | *Platelet-derived growth factor receptor beta (PDGFRB) (ENSMUSG00000024620)* | −2.66 (0.0162) | Positive regulator of angiogenesis |
| 25 | *Angiopoietin−1 receptor (TEK) (ENSMUSG00000006386)* | −2.7 (0.015) | Positive and negative regulator of angiogenesis |
| 26 | *fibulin 5 (Fbln5) (ENSMUSG00000021186)* | −2.72 (0.0014) | Endogenous inhibitor of angiogenesis |
| 27 | *Receptor-type tyrosine-protein phosphatase beta (PTPRB) (ENSMUSG00000020154)* | −2.72 (0.011) | Positive and negative regulator of angiogenesis |

| | | | |
|---|---|---|---|
| 28 | *collagen, type IV, alpha 1 (Col4a1) (ENSMUSG00000031502)* | −2.76 (0.016) | Inhibits angiogenesis and tumor formation |
| 29 | *fibronectin 1 (Fn1) (ENSMUSG00000026193)* | −2.83 (0.0283) | Inhibits angiogenesis and tumor formation |
| 30 | *Platelet-derived growth factor receptor alpha (PDG-FRA) (ENSMUSG00000029231)* | −2.91 (0.0001) | Promoter of angiogenesis |
| 31 | *Pigment epithelium-derived factor (SERPINF1) (ENSMUSG00000000753)* | −2.92 (0.0171) | Potent inhibitor of angiogenesis |
| 32 | *Sushi repeat-containing protein SRPX2 (SRPX2) (ENSMUSG00000031253)* | −3.1 (0.033) | Positive and negative regulator of angiogenesis |
| 33 | *Thrombospondin 2 (Thbs2) (ENSMUSG00000023885)* | −3.24 (0.0078) | Negative regulator of angiogenesis |
| 34 | *Vasohibin−1 (VASH1) (ENSMUSG00000021256)* | −3.34 (0.0316) | Angiogenesis inhibitor |
| 35 | *Platelet-derived growth factor D (PDGFD) (ENSMUSG00000032006)* | −3.5 (0.014) | Positive regulator of angiogenesis |
| 36 | *Protocadherin−12 (PCDH12) (ENSMUSG00000024440)* | −3.58 (0.03) | PCDH12 is required for normal angiogenesis and is highly expressed in angiogenic endothelial cells |
| 37 | *matrix metallopeptidase 2(Mmp2) (ENSMUSG00000031740)* | −4.27 (3.50E−05) | Positive and negative regulator of angiogenesis |
| 38 | *Coagulation factor XIII A chain (F13A1) (ENSMUSG00000039109)* | −4.39 (0.0000008) | Positive regulator of angiogenesis |
| 39 | *EGF-like repeats and discoidin I-like domains 3(Edil3) (ENSMUSG00000034488)* | −4.41 (0.0235) | Promotes angiogenesis |
| 40 | *Angiopoietin−4 (ANGPT4) (ENSMUSG00000027460)* | −5.16 (0.0003) | Promotes endothelial cell survival, migration and angiogenesis |
| 41 | *Angiopoietin−1 (ANGPT1) (ENSMUSG00000022309)* | −7.01 (0.0006) | Activates or inhibits angiogenesis, depending on the context |

Selected mouse genes from the down-regulated and up-regulated groups associated with angiogenesis. The proposed function indicated. For each gene, the gene symbol and name, fold change in expression and p-value are indicated. Negative numbers reflect down-regulation.

**Table S8.** VDAC1 silencing in a A549 lung cancer-derived tumor alters the expression of human ECM-related structural genes.

| No | Gene Name (Ensembl) | Fold change si-hVDAC1/si-NT (P value) | Proposed Function |
|---|---|---|---|
| | | **ECM-related structural genes** | |
| 1 | *Laminin subunit gamma−3 (LAMC3) (ENSMUSG00000043631)* | 7.46 (0.0061) | Structural molecule activity |
| 2 | *Laminin subunit alpha−1 (LAMA1) (ENSG00000101680)* | 5.25 (0.0059) | ECM structural component |
| 3 | *Fibroleukin (FGL2) (ENSG00000127951)* | 4.25 (0.0160) | ECM structural component |
| 4 | *Matrilin−3 (MATN3) (ENSG00000132031)* | −1.58 (0.0223) | Major component of cartilage and may play a role in the formation of extracellular filamentous networks |
| 5 | *Collagen alpha−2(V) chain (COL5A2) (ENSMUSG00000030116)* | −1.62 (0.0002) | Fibrillar-forming collagen |
| 6 | *Collagen alpha−1(V) chain (COL5A1) (ENSG00000130635)* | −1.67 (0.0001) | Fibrillar-forming collagen. |
| 7 | *Collagen alpha−1(XII) chain (COL12A1) (ENSG00000111799)* | −1.75 (0.0006) | ECM component conferring tensile strength |
| 8 | *Fibrillin−1 (FBN1) (ENSG00000166147)* | −1.83 (2.33E−05) | ECM component conferring elasticity |
| 9 | *Collagen alpha−2(IV) chain (COL4A2) (ENSG00000134871)* | −1.85 (0.0001) | ECM structural component |
| 10 | *Collagen alpha−1(IV) chain (COL4A1) (ENSG00000187498)* | −1.91 (4.09E−05) | ECM component conferring elasticity |
| 11 | *Collagen alpha−1(I) chain (COL1A1) (ENSG00000108821)* | −1.97 (0.0093) | Fibrillar-forming collagen |
| 12 | *Collagen alpha−2(I) chain (COL1A2) (ENSG00000164692)* | −2.09 (0.0153) | Fibrillar-forming collagen |

| | | | |
|---|---|---|---|
| 13 | *Collagen alpha−1(VIII) chain (COL8A1) (ENSG00000144810)* | −2.58 (0.0001) | Macromolecular component of the sub-endothelium |
| 14 | *Collagen alpha−1(XI) chain (COL11A1) (ENSG00000060718)* | −3.40 (0.0019) | Play an important role in fibrillogenesis by controlling lateral growth of collagen II fibrils |
| 15 | *Collagen alpha−2(VIII) chain (COL8A2) (ENSG00000171812)* | −4.53 (0.0001) | Macromolecular component of the sub-endothelium |

Selected human genes from the down-regulated and up-regulated groups associated with extracellular matrix structure. The proposed function indicated. For each gene, the gene symbol and name, fold change in expression and p-value are indicated. Negative numbers reflect down-regulation.

**Table S9.** VDAC1 silencing in A549 lung cancer-derived tumors alters the expression of human ECM organization genes.

| No | Gene Name (Ensembl) | Fold change si-hVDAC1/si-NT (P value) | Proposed Function |
|---|---|---|---|
| | | **ECM organization** | |
| 1 | *Heparan sulfate glucosamine 3-O-sulfotransferase 2 (HS3ST2) (ENSG00000122254)* | 7.16 (0.0336) | Sulfotransferase |
| 2 | *Inactive heparanase−2 (HPSE2) (ENSG00000172987)* | 5.73 (0.0077) | Binds heparin and heparan sulfate with high affinity but lacks heparanase activity. Inhibits HPSE |
| 3 | *Stabilin−2 (STAB2) (ENSG00000136011)* | 5.03 (0.0443) | Phosphatidylserine receptor that enhances the engulfment of apoptotic cells. Hyaluronan receptor that binds to and mediates endocytosis of hyaluronic acid |
| 4 | *LARGE xylosyl- and glucuronyltransferase 2 (GYLTL1B) (ENSG00000165905)* | 4.98 (0.0042) | Involved in protein glycosylation, a protein modification of ECM components necessary for ECM organization |
| 5 | *Heparanase (HPSE) (ENSG00000173083)* | 3.34 (0.0022) | Participates in ECM degradation and remodeling |
| 6 | *Type 2 lactosamine alpha−2,3-sialyltransferase (ST3GAL6) (ENSG00000064225)* | 2.69 (0.032) | Shows alpha−2,3-sialyltransferase activity toward Gal-beta(1,4)-GlcNAc on glycoproteins and glycolipids |
| 7 | *Perlecan (heparan sulfate proteoglycan 2) (HSPG2) (ENSG00000142798)* | −1.53 (0.0359) | An integral component of basement membranes where it is responsible for the fixed negative electrostatic charge and is involved in charge-selective ultrafiltration |
| 8 | *Lactosylceramide 1,3-N-acetyl-beta-D-glucosaminyl- transferase (B3GNT5) (ENSG00000176597)* | −1.63 (0.0364) | Plays a key role in the synthesis of lacto- or neolacto-series carbohydrate chains on glycolipids, notably by participating in the biosynthesis of HNK−1 and Lewis X carbohydrate structures |
| 9 | *Carbohydrate sulfotransferase 11 (CHST11) (ENSG00000171310)* | −1.64 (0.0186) | Catalyzes the transfer of sulfate to GalNAc position 4 in chondroitin |
| 10 | *Lysyl oxidase (LOX) (ENSG00000113083)* | −1.68 (0.0002) | Responsible for the post-translational oxidative deamination of peptidyl lysine residues in precursors to fibrous collagen and elastin |
| 11 | *Coiled-coil domain-containing protein 80 (CCDC80) (ENSG00000091986)* | −1.73 (2.3E−05) | Cell adhesion and matrix assembly |
| 12 | *Lysyl oxidase-like 2 (LOXL2) (ENSG00000134013)* | −1.79 (0.0155) | Promotes cross-linking of ECM proteins by mediating oxidative deamination of peptidyl lysine residues in precursors to fibrous collagen and elastin |
| 13 | *FERM domain-containing protein 5 (FRMD5) (ENSG00000171877)* | −1.80 0.0062 | Regulates cell-matrix interactions via its interaction with ITGB5. Cell migration |
| 14 | *Latent-transforming growth factor beta-binding protein 2 (Ltbp2) (ENSG00000119681)* | −1.81 (0.0009) | Plays an integral structural role in elastic-fiber architectural organization and/or assembly |
| 15 | *Mannose receptor C, type 2 (MRC2) (ENSMUSG00000020695)* | −1.83 (0.0073) | Internalizes glycosylated ligands, remodeling and degradation of extracellular collagen matrices |
| 16 | *Extracellular sulfatase Sulf−1 (SULF1) (ENSG00000137573)* | −1.97 (0.0290) | Arylsulfatase activity and highly specific endoglucosamine−6-sulfatase activity. Removes sulfate from the C−6 position of glucosamine in heparin. Diminishes heparan sulfate proteoglycan sulfation |
| 17 | *Biglycan (BGN) (ENSG00000182492)* | −2.06 (0.0065) | Involved in collagen fiber assembly |
| 18 | *Leucine-rich repeat-containing protein 15 (LRRC15) (ENSG00000172061)* | −3.2 (0.0019) | ECM protein binding |
| 19 | *Fibromodulin (FMOD) (ENSG00000122176)* | −3.92 (0.0013) | Affects the rate of fibrils formation. May play a primary role in collagen fibrillogenesis |
| 20 | *Cell migration-inducing and hyaluronan-binding protein (CE-MIP)(ENSMUSG00000030782)* | −3.93 ($1.63 \times 10^{-5}$) | Mediates depolymerization of hyaluronic acid via the cell membrane-associated clathrin-coated pit endocytic pathway. Binds to hyaluronic acid |

Selected human genes from the down-regulated and up-regulated groups associated with extracellular matrix organization. The proposed function indicated. For each gene, the gene symbol and name, fold change in expression and p-value are indicated. Negative numbers reflect down-regulation.

**Table S10.** VDAC1 silencing in A549 lung cancer-derived tumors alters the expression of human ECM peptidases genes.

| No | Gene Name (Ensembl) | Fold change si-hVDAC1/si-NT (P value) | Proposed Function |
|---|---|---|---|
| | | **ECM peptidases** | |
| 1 | *Macrophage metalloelastase (MMP12) (ENSG00000262406)* | 13.19 (0.0004) | Metalloelastase |
| 2 | *Disintegrin and metallo-proteinase domain-containing protein 33 (ADAM33) (ENSG00000149451)* | 5.92 (0.0063) | Metalloproteinase |
| 3 | *Dipeptidase 2 (DPEP2) (ENSG00000167261)* | 4.31 (0.028) | Probable metalloprotease which hydrolyzes leukotriene D4 into leukotriene E4. |
| 4 | *Pappalysin−1 (PAPPA) (ENSG00000182752)* | −1.57 (0.0182) | Metalloproteinase which specifically cleaves IGFBP−4 and IGFBP−5, resulting in release of bound IGF |
| 5 | *A disintegrin and metalloproteinase with thrombospondin motifs 15 (ADAMTS15)(ENSG00000166106)* | −1.95 (0.0068) | Metalloproteinase |
| 6 | *A disintegrin and metalloproteinase with thrombospondin motifs 4 (ADAMTS4)(ENSG00000158859)* | −1.97 (0.0185) | Metalloproteinase |
| 7 | *72 kDa type IV collagenase (MMP2) (ENSG00000087245)* | −2.09 (0.0053) | Collagenase |
| 8 | *A disintegrin and metalloproteinase with thrombospondin motifs 7 (ADAMTS7)(ENSG00000136378)* | −2.17 (0.0019) | Metalloproteinase |
| 9 | *Disintegrin and metalloproteinase domain-containing protein 19 (ADAM19) (ENSG00000135074)* | −2.30 (0.0045) | Protease |
| 10 | *Collagenase 3 (MMP13) (ENSG00000137745)* | −2.45 (0.0041) | Collagenase |
| 11 | *A disintegrin and metalloproteinase with thrombospondin motifs 12 (ADAMTS12) (ENSG00000151388)* | −3.93 (0.0012) | Metalloproteinase |
| 12 | *Matrix metalloproteinase−16 (MMP16) (ENSG00000156103)* | −3.96 ($2.98 \times 10^{-6}$) | Metalloproteinase |

Selected human genes from the down-regulated and up-regulated groups associated with extracellular matrix peptidases. The proposed function indicated. For each gene, the gene symbol and name, fold change in expression and p-value are indicated. Negative numbers reflect down-regulation.

**Table S11.** VDAC1 silencing in a A549 lung cancer-derived tumor alters the expression of human intercellular-interaction-associated genes.

| No | Gene Name (Ensembl) | Fold change si-hVDAC1/si-NT (P value) | Proposed Function |
|---|---|---|---|
| | | **Human intercellular-interaction-associated genes** | |
| 1 | *Cadherin−3 (CDH3) (ENSG00000062038)* | 7.61 (0.0004) | Calcium-dependent cell adhesion protein |
| 2 | *Down syndrome cell adhesion molecule (DSCAM) (ENSG00000171587)* | 5.69 (0.043) | Mediates intracellular signaling by stimulating the activation of MAPK8 and MAP kinase p38. Adhesion molecule that promotes lamina-specific synaptic connections in the retina |
| 3 | *Protocadherin−7 (PCDH7) (ENSG00000169851)* | 5.03 (0.004) | Homophilic cell adhesion via plasma membrane adhesion molecules. Platelet degranulation |
| 4 | *Tyrosine-protein kinase Mer (MERTK) (ENSG00000153208)* | 4.31 (0.008) | Plays a role in various processes, such as macrophage clearance of apoptotic cells, platelet aggregation, cytoskeleton reorganization and engulfment. Induces production of suppressors of cytokine signaling SOCS1 and SOCS3 |

| | | | |
|---|---|---|---|
| 5 | *Homeobox protein DLX−1 (DLX1) (ENSG00000144355)* | 3.26 (0.034) | Inhibits several cytokine signaling pathways, such as TGFB1, activin-A/INHBA and BMP4 by interfering with the transcriptional stimulatory activity of transcription factors, such as MSX2, FAST2, SMAD2 and SMAD3 during hematopoietic cell differentiation |
| 6 | *Fermitin family homolog 3 (FERMT3) (ENSG00000149781)* | 2.43 (0.011) | Plays a central role in cell adhesion. Required for activation of integrin beta−2 in polymorphonuclear granulocytes |
| 7 | *Armadillo repeat protein deleted in velo-cardio-facial syndrome (ARVCF) (ENSG00000099889)* | 2.4 (0.033) | Involved in protein-protein interactions at adherens junctions |
| 8 | *Desmoplakin (DSP) (ENSG00000096696)* | 2.24 (0.033) | Major high molecular weight protein of desmosomes. Involved in the organization of the desmosomal cadherin-plakoglobin complexes into discrete plasma membrane domains and in the anchoring of intermediate filaments to the desmosomes |
| 9 | *Arf-GAP with coiled-coil, ANK repeat and PH domain-containing protein 1 (ACAP1) (ENSG00000072818)* | 1.79 (0.0007) | Required for regulated export of ITGB1 from recycling endosomes to the cell surface and ITGB1-dependent cell migration |
| 10 | *N-acetylneuraminate lyase (NPL) (ENSG00000135838)* | 1.63 (0.045) | Catalyzes the cleavage of N-acetylneuraminic acid to form pyruvate and N-acetylmannosamine. Prevents sialic acids from being recycled to the cell surface |
| 11 | *Arylsulfatase J (ARSJ) (ENSG00000180801)* | −1.51 (0.023) | Glycosphingolipid metabolic process, post-translational protein modification |
| 12 | *Blood vessel epicardial substance (BVES) (ENSG00000112276)* | −1.55 (0.0151) | Cell adhesion molecule involved in the establishment and/or maintenance of cell integrity. Involved in the formation and regulation of the tight junction paracellular permeability barriers in epithelial cells |
| 13 | *Discoidin, CUB and LCCL domain-containing protein 2 (DCBLD2)(ENSG00000057019)* | −1.55 (0.027) | Negative regulator of cell growth |
| 14 | *Pleckstrin homology-like domain family B member 2 (PHLDB2) (ENSG00000144824)* | −1.56 (0.0044) | Cadherin binding |
| 15 | *Sushi, von Willebrand factor type A, EGF and pentraxin domain-containing protein 1 (SVEP1) (ENSG00000165124)* | −1.64 (0.0157) | Plays a role in the cell attachment process |
| 16 | *Protein NOV homolog (NOV) (ENSG00000136999)* | −1.68 (0.0397) | Fibroblast adhesion through ITGA5:ITGB1 and ITGA6:ITGB1 and induces fibroblast chemotaxis through ITGAV:ITGB5 |
| 17 | *Anthrax toxin receptor 1 (ANTXR1) (ENSG00000169604)* | −1.70 (0.0470) | Cell attachment via collagen 1 and gelatin, migration. Interacts with ECM proteins |
| 18 | *Cadherin−4 (CDH4) (ENSG00000179242)* | −1.73 (0.0483) | Cell adhesion protein |
| 19 | *Cadherin−2 (CDH2) (NCAD) (ENSG00000170558)* | −1.76 (0.0057) | Cell adhesion protein |
| 20 | *Cadherin−5 (CDH5) (ENSG00000179776)* | −2.36 (0.0134) | Cell adhesion protein |
| 21 | *Probable carboxypeptidase X1 (CPXM1) (ENSG00000088882)* | −2.76 (0.0025) | May be involved in cell-cell interactions |
| 22 | *Integrin alpha-D (ITGAD) (ENSG00000156886)* | 4.33 (0.0050) | Alpha subunit of the cell surface heterodimer. Involved in the activation and adhesion functions of leukocytes |
| 23 | *Integrin beta (ITGB2) (ENSG00000160255)* | 3.03 (0.0020) | Receptor for ICAM1, ICAM2, ICAM3 and ICAM4 |
| 24 | *Integrin alpha-IIb (ITGA2B) (ENSG00000005961)* | 2.69 (0.0263) | Receptor for fibronectin, fibrinogen, plasminogen, prothrombin, thrombospondin and vitronectin |
| 25 | *Integrin beta-like protein 1 (ITGBL1) (ENSG00000198542)* | −2.35 (0.0416) | Cell adhesion protein |
| 26 | *Integrin alpha−11 (ITGA11) (ENSG00000137809)* | −2.61 (0.0018) | Receptor for collagen |
| 27 | *Integrin binding sialoprotein (IBSP) (ENSG00000029559)* | −10.73 (0.0110) | Appears to form an integral part of the mineralized matrix. Probably important for cell-matrix interaction |
| 28 | *Intercellular adhesion molecule 5 (ICAM5) (ENSG00000105376)* | 5.48 (0.0054) | Integrin binding |
| 29 | *Neuronal cell adhesion molecule (NRCAM) (ENSG00000091129)* | −2.55 (0.0072) | Cell adhesion protein that is required for normal responses to cell-cell contacts in brain and the peripheral nervous system |
| 30 | *Claudin−11 (CLDN11) (ENSG00000013297)* | 2.35 (0.0463) | Plays a major role in tight junction-specific obliteration of the intercellular space |

| | | | |
|---|---|---|---|
| 31 | *Chymotrypsin-like elastase family member 1 (CELA1) (ENSG00000139610)* | −4.06 (0.0447) | Acts on elastin |
| 32 | *Endomucin (EMCN) (ENSG00000164035)* | −7.95 (0.0236) | Interferes with the assembly of focal adhesion complexes and inhibits interaction between cells and the ECM |
| 33 | *Insulin growth factor-like family member 2 (IGFL2) (ENSG00000204866)* | −4.17 (0.0011) | Potential ligand of the IGFLR1 cell membrane receptor |

Selected human genes from the down-regulated and up-regulated groups associated with intercellular-interaction. The proposed function indicated. For each gene, the gene symbol and name, fold change in expression and p-value are indicated. Negative numbers reflect down-regulation.

**Table S12.** VDAC1 silencing in a A549 lung cancer-derived tumor alters the expression of human angiogenesis-associated genes.

| No | Gene Name (Ensembl) | Fold change si-hVDAC1/si-NT (P value) | Proposed Function |
|---|---|---|---|
| | | **Angiogenesis** | |
| 1 | *Ectonucleotide pyrophosphatase/phosphodiesterase family member 2 (ENPP2) (ENSG00000136960)* | 16.69 (0.00001) | Acts as an angiogenic factor by stimulating migration of smooth muscle cells and microtubule formation. Stimulates migration of melanoma cells, probably via a pertussis toxin-sensitive G protein. May play a role in induction of parturition. Possible involvement in cell proliferation and adipose tissue development. Tumor cell motility-stimulating factor |
| 2 | *Receptor-type tyrosine-protein kinase FLT3 (FLT3) (ENSG00000122025)* | 10.96 (0.0056) | Activated receptor kinase subsequently phosphorylates and activates multiple cytoplasmic effector molecules in pathways involved in apoptosis, proliferation, and differentiation of hematopoietic cells in bone marrow. Mutations that result in the constitutive activation of this receptor result in acute myeloid leukemia and acute lymphoblastic leukemia |
| 3 | *Phosphoinositide 3-kinase regulatory subunit 6 (PIK3R6) (ENSG00000276231)* | 4.82 (0.024) | Positive angiogenesis regulation. Regulatory subunit of the PI3K |
| 4 | *Filamin A-interacting protein 1-like (FILP1L) (ENSG00000168386)* | 4.75 (0.0027) | Acts as a regulator of anti-angiogenic activity on endothelial cells. When overexpressed in endothelial cells, leads to inhibition of cell proliferation and migration and increased apoptosis. Inhibits melanoma growth when expressed in tumor-associated vasculature |
| 5 | *Secretogranin−2 (SCG2) (ENSG00000171951)* | 4.52 (0.018) | Neuroendocrine secretory granule protein. Precursor for biologically active peptides. Positive regulator of angiogenesis |
| 6 | *Sema domain, immuno-globulin domain (Ig), transmembrane domain (TM) and short cytoplasmic domain (semaphorin) 4A(SEMA4A) (ENSMUSG00000028064)* | 3.9 (0.014) | Negative regulator of angiogenesis |
| 7 | *Ephrin-A3 (EFNA3) (ENSG00000143590)* | 2.21 (0.04) | Negative regulator of angiogenesis |
| 8 | *Ras-interacting protein 1 (RASIP1) (ENSG00000105538)* | 2.18 (0.041) | Required for proper formation of vascular structures that develop via both vasculogenesis and angiogenesis. Acts as a critical and vascular-specific regulator of GTPase signaling, cell architecture, and adhesion, which is essential for endothelial cell morphogenesis and blood vessel tubulogenesis |
| 9 | *Guanine nucleotide exchange factor VAV3 (VAV3)(ENSG00000134215)* | 2 (0.018) | Plays an important role in angiogenesis |
| 10 | *Angio-associated migratory cell protein (AAMP) (ENSG00000127837)* | 1.51 (0.0214) | Member of the immunoglobulin superfamily. Associated with angiogenesis, with potential roles in endothelial tube formation and migration of endothelial cells |
| 11 | *Platelet-derived growth factor C (PDGFC) (ENSG00000145431)* | −1.68 (0.0007) | Pro-angiogenic factor |
| 12 | *Platelet-derived growth factor receptor beta (PDGFRB) (ENSG00000113721)* | −1.72 (0.0130) | Pro-angiogenic factor |
| 13 | *R-spondin−3 (RSPO3) (ENSG00000146374)* | −1.84 (0.0331) | Activator of the Wnt signaling pathway by acting as a ligand for LGR4−6 receptors, key regulators of angiogenesis |
| 14 | *Sushi repeat-containing protein SRPX2 (SRPX2) (ENSG00000102359)* | −2.14 (0.0143) | Plays a positive role in angiogenesis by inducing endothelial cell migration and the formation of vascular network (cords). Involved in cellular migration and adhesion |
| 15 | *Hedgehog-interacting protein (HHIP) (ENSG00000164161)* | −2.21 (0.0168) | Negative regulatior of angiogenesis |

| | | | |
|---|---|---|---|
| 16 | *UPF0606 protein KIAA1549L (KIAA1549L) (ENSG00000110427)* | −2.71 (0.0100) | Development VEGF signaling via VEGFR2-generic cascades. Angiogenesis |
| 17 | *Apelin receptor (APLNR) (ENSG00000134817)* | −2.76 (0.0197) | Pro-angiogenic factor |

Selected human genes from the down-regulated and up-regulated groups associated with angiogenesis. The proposed function indicated. For each gene, the gene symbol and name, fold change in expression and p-value are indicated. Negative numbers reflect down-regulation.

**Table S13.** VDAC1 silencing in a A549 lung cancer-derived tumor alters the expression of human growth factor genes.

| **No** | **Gene Name (Ensembl)** | **Fold change si-hVDAC1/si-NT (P value)** | **Proposed Function** |
|---|---|---|---|
| | | **Growth factors** | |
| 1 | *Fibroblast growth factor 5 (FGF5) (ENSG00000138675)* | 11.88 (0.0088) | Plays important role in regulating cell proliferation and cell differentiation |
| 2 | *Fibroblast growth factor 8 (FGF8) (ENSG00000107831)* | 4.78 (0.0019) | Plays an important role in regulating embryonic development, cell proliferation, cell differentiation and cell migration |
| 3 | *Transforming growth factor-beta-induced protein ig-h3 (TGFBI) (ENSG00000120708)* | −1.61 (0.0276) | Plays a role in cell adhesion. May play a role in cell-collagen interactions, angiogenesis |
| 4 | *Fibroblast growth factor 12 (FGF12) (ENSG00000114279)* | −1.68 (0.0198) | Involved in the positive regulation of voltage-gated sodium channel activity |
| 5 | *Transforming growth factor, beta 2 (TGFB2) (ENSG00000092969)* | −2.03 (1.52E−07) | Has suppressive effects on interleukin−2-dependent T-cell growth. |
| 6 | *Insulin growth factor-like family member 1 (IGFL1) (ENSG00000188293)* | −2.94 (0.0037) | Probable ligand of the IGFLR1 cell membrane receptor |
| 7 | *Insulin growth factor-like family member 2 (IGFL2) (ENSG00000204866)* | −4.17 (0.0011) | Potential ligand of the IGFLR1 cell membrane receptor |

Selected human genes from the down-regulated and up-regulated groups associated with growth factors. The proposed function indicated. For each gene, the gene symbol and name, fold change in expression and p-value are indicated. Negative numbers reflect down-regulation.

**Publisher's Note:** MDPI stays neutral with regard to jurisdictional claims in published maps and institutional affiliations.

© 2021 by the authors. Licensee MDPI, Basel, Switzerland. This article is an open access article distributed under the terms and conditions of the Creative Commons Attribution (CC BY) license (http://creativecommons.org/licenses/by/4.0/).
